# Regulation of tumor immunity and immunotherapy by the tumor collagen extracellular matrix

**DOI:** 10.3389/fimmu.2023.1199513

**Published:** 2023-08-17

**Authors:** Dallas B. Flies, Solomon Langermann, Christina Jensen, Morten A. Karsdal, Nicholas Willumsen

**Affiliations:** ^1^ NextCure Inc., Beltsville, MD, United States; ^2^ Nordic Bioscience A/S, Herlev, Denmark

**Keywords:** cancer biology, collagen, cancer immunotherapy, ECM - extracellular matrix, LAIR-1, tumor microenvironment (TME)

## Abstract

It has been known for decades that the tumor extracellular matrix (ECM) is dysfunctional leading to loss of tissue architecture and promotion of tumor growth. The altered ECM and tumor fibrogenesis leads to tissue stiffness that act as a physical barrier to immune cell infiltration into the tumor microenvironment (TME). It is becoming increasingly clear that the ECM plays important roles in tumor immune responses. A growing body of data now indicates that ECM components also play a more active role in immune regulation when dysregulated ECM components act as ligands to interact with receptors on immune cells to inhibit immune cell subpopulations in the TME. In addition, immunotherapies such as checkpoint inhibitors that are approved to treat cancer are often hindered by ECM changes. In this review we highlight the ways by which ECM alterations affect and regulate immunity in cancer. More specifically, how collagens and major ECM components, suppress immunity in the complex TME. Finally, we will review how our increased understanding of immune and immunotherapy regulation by the ECM is leading towards novel disruptive strategies to overcome immune suppression.

## 1 Introduction

**Figure 1:**
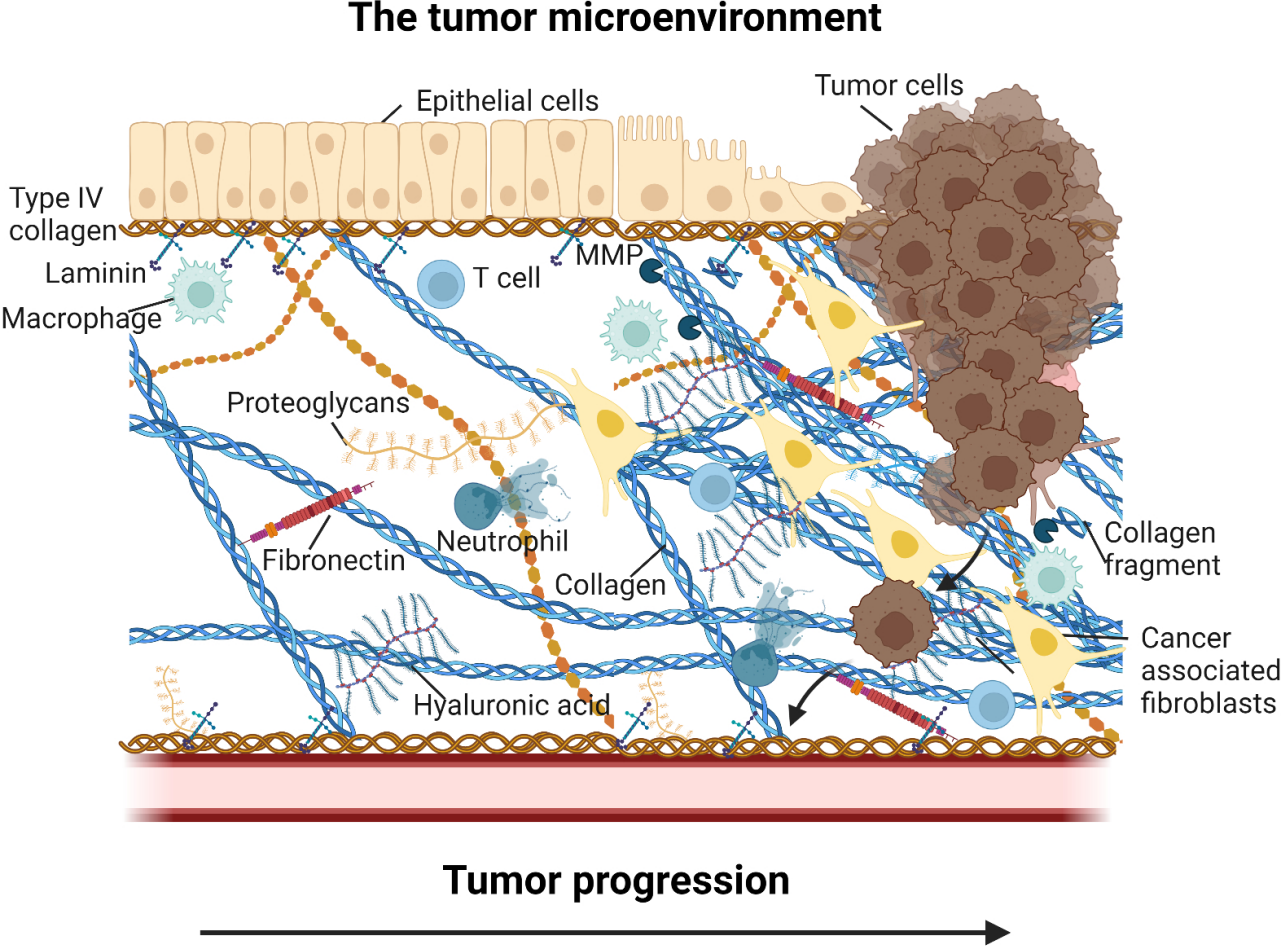
Overview of the tumor microenvironment (TME). In addition to the tumor cells, the TME consist of non-malignant stromal cells such as cancer associated fibroblasts, immune cells such as tumor associated macrophages, tumor associated neutrophils, T cells, secreted factors such proteases, cytokines and growth factors, and the tumor extracellular matrix (ECM). The ECM include core components such as collagens, fibronectin, hyaluronan, and laminins, and a wide array of proteoglycans and associated molecules. Tumor ECM changes may alter ECM-cell interactions and thereby prevent T cell recruitment to the tumor cells (immune exclusion) and drive immune reprogramming and modulation of immune cell activity (immune suppression) supporting tumor progression and lead to poor efficacy of intervention. Figure created with BioRender.com.

Tumors consists of cancer cells and their immediate environment, the tumor microenvironment (TME) (1). The TME is a heterogeneous amalgamation of non-malignant stromal cells, immune cells, secreted factors, and the tumor extracellular matrix (ECM). An overview of the TME is shown in **Figure 1**. It is now established that for anti-cancer treatment to be successful, therapeutics needs to not only eradicate the cancer cells, but it is equally important also target the TME, for example by modulating stromal cell activity, immune cell activity and phenotype, and by interfering with ECM-cell receptor interactions (2–7). Reprogramming of the immune response from pro-tumorigenic to anti-tumorigenic is an essential component for therapeutic success and understanding the drivers of an immunosuppressive environment will help advance the field (8). It is now evident that the ECM is a major player in facilitating both tumor progression and resistance to various treatments, including immunotherapy (9–13).

Research on the regulation of tumor immunity by the tumor ECM is rapidly expanding. Historically, the tumor ECM has largely been associated with a physical barrier that excludes immune cell access to the tumor. An expanding concept is that ligands from the ECM and its constituent components have direct, active effects on immune cells by binding to receptors that either stimulate or inhibit signaling pathways, and thus play a more active role in cancer immunosurveillance.

Under physiological conditions the ECM forms a proteinaceous network between cells in the tissue and thereby contributes to the arrangement and polarity of cells that support their survival and differentiation and maintain tissue organization (14). The ECM consists of a complex network of macromolecules including collagens, elastin, fibronectin, laminins, proteoglycans, and non-collagenous glycoproteins. Collagens are the major components of the ECM. Twenty-eight different types of collagen have been described, each with a unique role in maintaining tissues structure and function (15).

ECM biology has been a major area of focus in cancer research for well over forty years and aberrant production of ECM constituents is a classic hallmark of cancer progression (10, 16, 17). A wealth of knowledge has been developed that continues to build on our understanding of just how dramatically different cancer ECM is from normal EC. This includes the mechanisms and biophysics that lead to dramatic alterations seen in cancer (18, 19). What has been termed the Matrisome in cancer describes ECM proteins, particularly collagens, that are not only overexpressed, but structurally and biochemically aberrant from normal, healthy tissues (12, 20–22).

Most ECM biology studies in cancer have focused on understanding how the ECM modifies tumor cell transformation, growth, movement and metastasis, with less attention paid to its role in immune surveillance (23). The tools for understanding tumor immunology and developing novel immunotherapies has exploded over the past thirty years, largely due to the now well-established theory of tumor immune surveillance (24). A key aspect of tumor immune surveillance is that costimulatory or coinhibitory ligands, such as B7-1 or PD-L1, that interact with cognate costimulatory or coinhibitory receptors, such as CD28/CTLA-4 or PD-1, on T cells to elicit or suppress tumor-antigen-specific immune responses, respectively (25). These types of ligand-receptor interactions have a direct, active effect on immune cells mediated by downstream signaling. These studies have been fruitful regarding the development of immune checkpoint inhibitors targeting PD-1/PD-L1, CTLA-4, LAG-3 and other emerging therapeutics targeting molecules expressed on immune subpopulations (26). There is abundant literature that not only T cells, but other immune subpopulations such as NK cells, B cells, macrophages, dendritic cells (DCs) and neutrophils are critical to immune surveillance (reference 23 and new reference Shi et al). Tumors subvert the function of multiple cell types to cause immune dysfunction and immune suppression that ultimately leads to immune suppression in the complex TME. Therapeutics that target these immune subpopulations are emerging as potential next-generation immune therapies beyond checkpoint inhibitors (27).

Until recently, much of the work in tumor immune surveillance has focused on identifying and characterizing inter-cellular immune ligand-receptor interactions, with little attention to the ECM as a source of ligands and epitopes with either immune-activating and/or immune-suppressive signaling capacity, depending on the architecture and quality of the ECM. However, the fact that the ECM may be a storage of ligands and epitopes with signaling capacity has been known for decades. There are several hundred molecules that make up the core matrisome and associated ECM constituents (20). The overall composition and quality of core components and associated constituents are vastly altered in cancer (12). Continuing research and new technologies continue to define and elucidate the wealth of ligands and epitopes that may play a role in cancer immune surveillance and could be potentially used as cancer biomarkers and be targeted for cancer immunotherapy.

While it is not the purpose of this review to address all components of the ECM, collagens are particularly well described beyond their function as passive, structural molecules in the TME, and are now being recognized for their active contribution to several biological effects in the TME. Of the 28 types of collagen, collagens type IV, XIII, XV, and XVIII have received the most attention in the cancer field, consequent to the anti-angiogenic and pro-tumorigenic effects of the cryptic sites and signaling fragments found in the NC1 domains (28). The best characterized basement membrane collagen signals are derived from type IV collagen (Arresten, Canstatin, Tumstatin, Tetrastatin, Pentastatin, Hexastatin), type VIII collagen (Vastatin), type XV collagen (Restin), and type XVIII collagen (Endostatin) (29). Endothrophin, a signaling fragment derived from type VI collagen produced by fibroblasts is receiving increased attention in cancer and other fields where fibroblasts are central players (29, 30). The biological role of endothrophin in cancer is related to epithelial-to-mesenchymal transition and tumor fibrosis. Endothrophin is highly expressed in CAFs and is prognostic in a range of fibrotic diseases, including liver, lung, kidney, and skin fibrosis (31–36). Understanding how collagens and collagen fragments actively bind and regulate immune receptors, in addition to functions described here, will be critical to link cancer matrix biology and immune oncology.

Strides are being made in understanding how ECM components actively regulate immune cellular exclusion, activation, and suppression in cancer. Consequently, targeting the tumor promoting effects of the dysfunctional ECM is a novel approach to cancer immunotherapy. Building on recent understanding of the ECM-immune cell interplay will help overcome current limitations in cancer immunotherapy. Recent studies and concepts have started to combine these fields to identify novel links and biological understanding of these interactive pathways that will help lead the next wave of cancer immunotherapies.

## 2 Tumor ECM acts as a physical barrier to immune cell infiltration

The altered production and assembly of ECM proteins and collagens generally forms a fibrous connective tissue by interacting with other ECM components and is the major pathological signature of tumor fibrogenesis (also known as desmoplasia). Cross-linking of collagens by lysyl oxidase (LOX) enzymes increases the stiffness of this ECM (37). Transforming growth factor beta (TGF-β) and other pro-fibrotic cytokines signal to fibroblast and activate them into cancer associated fibroblasts (CAFs) with associated increased collagen synthesis (38–41). During cancer, an accumulation of activated CAFs is observed (42). Enhanced CAF activity results in increased deposition of a cross-linked collagen matrix in the TME (43, 44). CAF subtypes is the center of a lot of attention currently and a detailed description is beyond the scope of this review. However, evidence shows that some CAF subsets promote tumor progression and immunosuppression, while others prevent it (45–48), but the overall consensus is that the fibroblasts drive fibrosis and tumor progression (29). In fact, several recent studies suggest that alterations in ECM proteins, fibrillar collagens, CAFs, and increased expression of TGF-β all contribute to fibrosis and play key roles in resistance to immunotherapy by creating a physical barrier inhibiting T cell infiltration (immune exclusion) that is crucial for anti-tumor immunity and concomitant clinical responses to current checkpoint inhibitors (49–61).

## 3 Tumor ECM ligands interact with immune cell receptors

Beyond the dense and fibrotic tumor ECM barrier associated with immune exclusion, tumor ECM components also play a more active role in immune regulation when dysregulated ECM components act as ligands to interact with receptors on immune cells to inhibit or activate immune cell subpopulations in the TME. Thus, an expanding paradigm is that ECM-derived molecules are capable of interacting with and regulating immune cells not only in the context of well-described adhesive binding interactions and barrier function, but also through active interactions with immune cell inhibitory or stimulatory receptors to modulate T cells, myeloid cells and other immune cell types in the TME (62).

A primary mechanism of T cell suppression is through interaction of T cell surface inhibitory receptors with inhibitory ligands expressed on the cell surface of tumor cells, tumor associated macrophages (TAMs) and DCs (TADCs), myeloid-derived suppressor cells (MDSCs) or other suppressive cell types in the TME (63). Under normal conditions, inhibitory cell-cell interactions between receptors and ligands serve as a means of communication to maintain T cell tolerance to self, and to avoid dangerous autoreactive immune responses leading to autoimmunity. In the TME, aberrant expression of these receptors and ligands interact to circumvent anti-tumor T cell immunity. Blockade of cell-cell interaction-mediated immune inhibition is the basis of immune checkpoint inhibitor (ICI) immunotherapies that promote T cell anti-tumor immunity mediated by tumor-specific T cells. In addition to cell-cell molecular interactions, what is becoming more apparent is that ECM proteins can also function as inhibitory and stimulatory ligands to disrupt T cell and myeloid responses in the TME. T cells expressing inhibitory receptors, often defined as exhausted T cells, and myeloid cells - TAMs, TADCs and MDSCs – that have a suppressive phenotype, and are generally associated with poor prognosis in most cancers. There may be many reasons why tumor associated T cells and myeloid cells develop suppressive activity, but studies now suggest T cell and myeloid cell interaction with the ECM, including collagens, and mechanical properties such as collagen density and stiffness, contribute to direct suppression of immune function, or polarization of cells towards a suppressive phenotype and facilitate recruitment of suppressive cells into the TME (64, 65).

The immune regulatory function of the tumor ECM seems to be confined to specific ECM receptors, of which integrins and growth factor receptors are well known ECM binding receptors (reviewed in (66–69)). More recently, greater attention has been focused on an emerging set of regulatory receptors specifically expressed on immune cells that interact with ECM proteins to directly regulate immune function. The broad dysregulation of collagen and other ECM proteins that occurs in the TME can interact with aberrant expression of these inhibitory and stimulatory immune receptors to drive immune dysfunction in cancer. Emerging receptors in this context include LAIR-1, OSCAR and DDR1/DDR2, that are expressed on immune cells and interact with collagens to regulate immune function. Another receptors emerging in this context is LILRB4, a protein that interacts with fibronectin, a non-collagen component of the ECM CD44 and Toll-Like Receptors (TLRs), which binds more promiscuously ECM and non-ECM ligands.

### 3.1 ECM receptors that regulate tumor immunity

**Figure 2:**
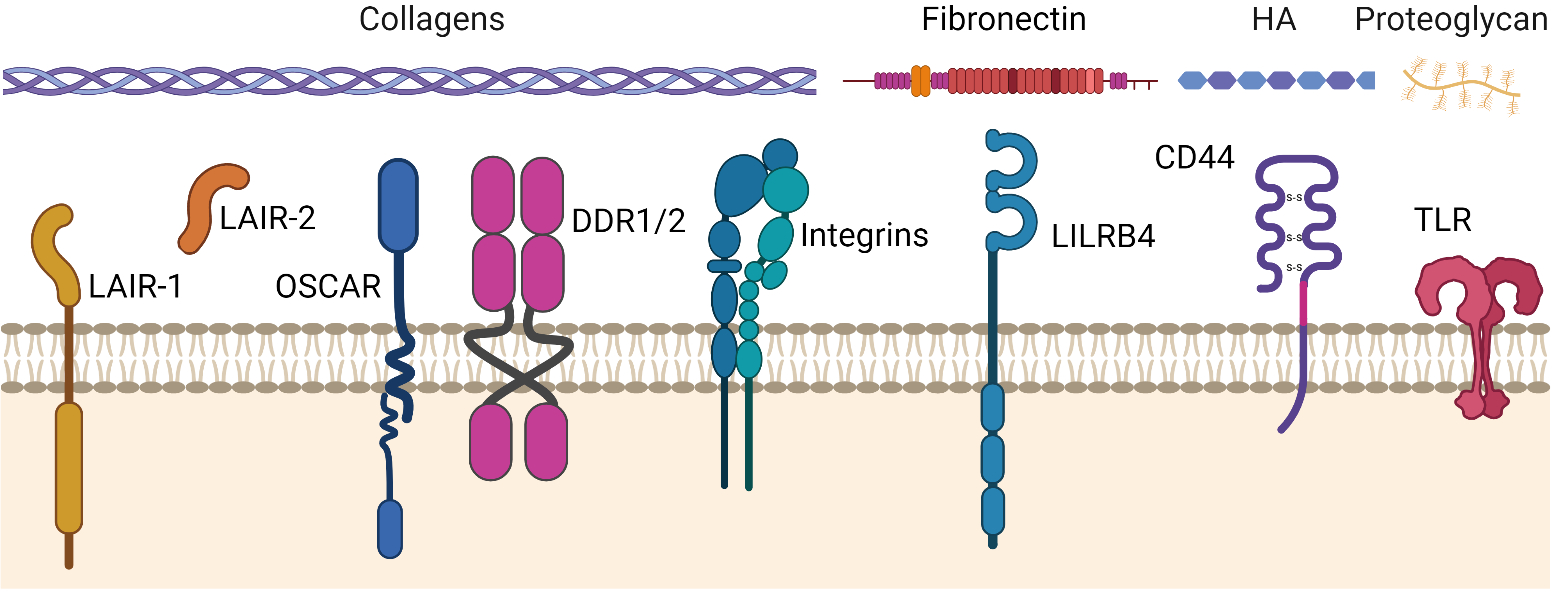
ECM receptors that may regulate tumor immunity in cancers. ECM receptors and their primary ligands (collagens, fibronectin, hyaluronic acid (HA), proteoglycans). The ECM stimulates or inhibits cellular activity through these receptors in response to, and dependent on, an injured, remodeled, or dysregulated ECM composition. LAIR-1, Leukocyte-Associated Immunoglobulin-like Receptor-1. LAIR-2, Leukocyte-Associated Immunoglobulin-like Receptor-2. OSCAR, Osteoclast-associated receptor. DDR-1/2, Discoidin Domain Receptors 1 and 2. LILRB4, Leukocyte immunoglobulin-like receptor subfamily B member 4 (a.k.a. ILT3). CD44, Cluster of differentiation 44. TLR, Toll-Like Receptor. Figure created with BioRender.com.

An overview of the ECM receptors that regulate tumor immunity is shown in **Figure 2** and summarized below.

#### 3.1.1 Integrins

Integrins are so-called heterodimeric receptors that are composed of α and β subunits (there are eight β and 18 α subunits in the integrin family that combine to form at least 24 distinct integrins). Integrins are cell adhesion receptors that play important roles during pathological processes and development (66). Integrins are transmembrane proteins composed of a short cytoplasmic region mediating the downstream signaling from the receptor, a transmembrane helix, and a large extracellular domain. The 24 distinct integrins are divided into four classes (RGD receptors, leukocyte-specific receptors, laminin receptors, and collagens receptors). The integrins that function primarily as collagen receptors are α1β1, α2β1, α3β1, α10β1 and α11β1 (67). The interplay between integrins and immune cells for cancer immunity and the role of integrin receptor interactions with the TME-ECM and targeting for cancer therapy is beyond the scope of this review and has been reviewed elsewhere (68, 69).

#### 3.1.2 LAIR-1

**Figure 3:**
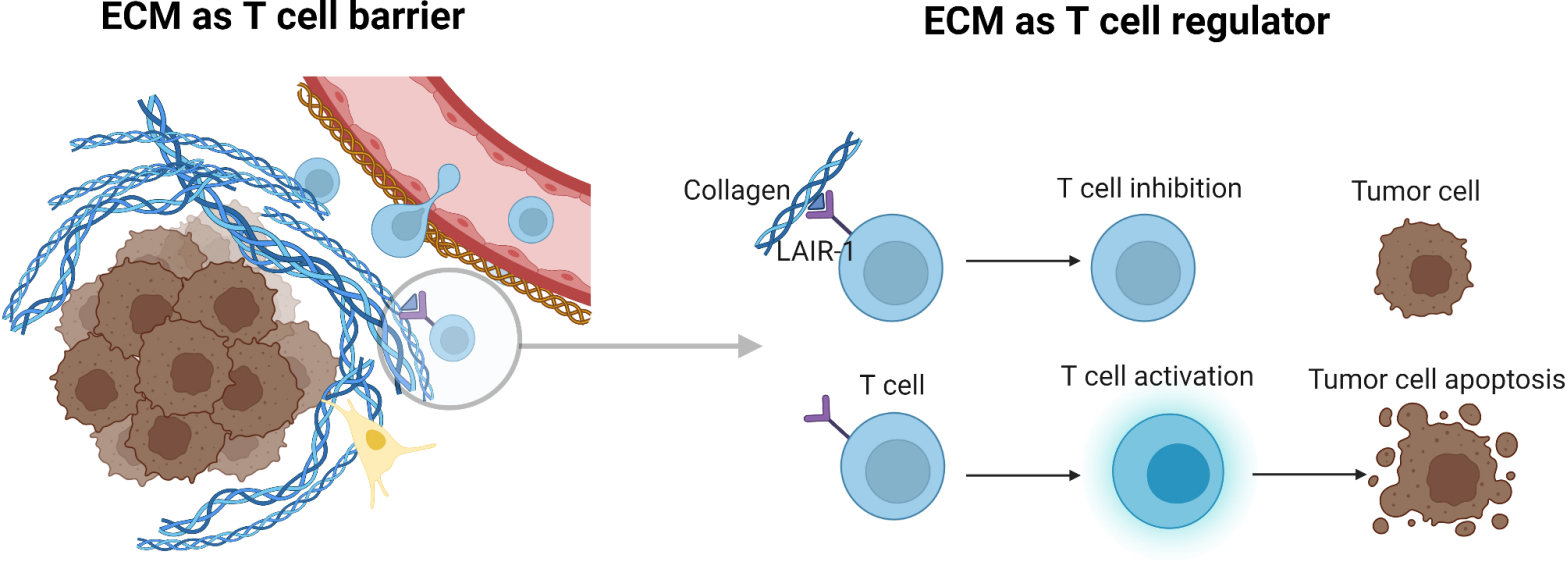
Tumor ECM as a T cell barrier and regulator of T cell activity. Aberrant collagen expression may both exclude immune cells from infiltrating the TME, and at the same time prevent T cells from becoming activated and developing into cytotoxic effector T cells. LAIR-1 as an example plays both a role in cellular adhesion to the ECM, and at the same time delivers signals to instruct immune cells to remain in a sub-optimal state of activation. Figure created with BioRender.com.

The coinhibitory Leukocyte-Associated Immunoglobulin-like Receptor-1 (LAIR-1) is type-I transmembrane receptor expressed on T cells, NK cells, myeloid cells and other immune cell subsets, and binds to collagens and proteins with collagen-like domains (70). LAIR-1 expression is also abundant on TME immune cells and may increase with stage of disease (71–73). LAIR-1 contains motifs in its cytoplasmic region, including immunoreceptor tyrosine-based inhibitory motifs (ITIMs) and (CSK) that induce inhibitory signaling pathways into cells when LAIR-1 binds to collagen domain containing ligands (74, 75). As such, LAIR-1 interaction with collagens plays both a role in cellular adhesion to the ECM, and at the same time delivers signals to instruct immune cells to remain in a sub-optimal state of activation. Interestingly, under normal physiological conditions, LAIR-1 may play a limited role in maintaining immune homeostasis (76). However, when the ECM becomes dysfunctional in TMEs, aberrant collagen expression may both exclude LAIR-1 expressing immune cells from infiltrating the TME, and at the same time prevent tumor antigen-specific T cells from becoming activated and developing into cytotoxic effector T cells through LAIR-1 mediated inhibitory signaling (55, 72, 77, 78) (**Figure 3**). Expression of LAIR-1 on myeloid cells in cancer has been shown to play a role in immune suppression, also mediated primarily through collagen (79). In contrast, another study suggested an improved response in a pre-clinical model when LAIR-1 was present on myeloid cells (80). However, the observed function was attributed to interaction with a collagen-domain containing protein, COLEC12, rather than structural collagen of the ECM. Additional understanding of collagen-LAIR-1 mediated regulation of T cells and myeloid cells will be an important area of research for therapeutic targeting in both solid tumors and hematologic malignancies.

#### 3.1.3 DDR1 and DDR2

Discoidin Domain Receptors 1 and 2 (DDR1 and DDR2) are receptor tyrosine kinases (RTKs) that uniquely bind to fibrillar collagens; DDR1 preferentially binds to collagen I–V, VIII (and Periostin), while DDR2 binds to collagen I–III, V, and X (81, 82). Indeed, studies have identified multiple effects of DDR1 and DDR2 on tumor growth and metastasis when expressed on tumor and CAFs (9, 83). DDR2 is also expressed on subpopulations of tumor myeloid cells and drives myeloid inflammatory pathways *via* RTK signaling (83). The composition and quality of the ECM is important for DDR signaling. For example, protease-cleaved type I collagen and intact type I collagen were shown to have opposing tumorigenic effects through DDR1 engagement in pancreatic cancer (84). Dissection of the role of DDR1/2 in immune regulation will shed light on the role of these important collagen receptors in cancer.

#### 3.1.4 OSCAR

The Osteoclast-associated receptor (OSCAR) is a collagen receptor in the same family as LAIR-1, but with stimulatory signaling capacity through cytoplasmic association with FcRγ, which contains an Immunoreceptor Tyrosine-based Activation Motif (ITAM) (85, 86). OSCAR is expressed not only on osteoclasts, but also on other myeloid cell subsets, and OSCAR RNA is overexpressed in several cancers (87). Interestingly, despite OSCAR’s stimulatory capacity, RNA expression appears to positively associate with M2 macrophage differentiation, T cell exhaustion, cancer progression and metastasis, although much remains to be learned in the context of cancer.

#### 3.1.5 LILRB4

Leukocyte immunoglobulin-like receptor subfamily B member 4 (LILRB4/ILT3) is an inhibitory ITIM containing Ig-superfamily and LILR family receptor that is expressed on DCs and other myeloid cells that binds to fibronectin (85, 88). In a recent study it was shown that LILRB4 interactions with fibronectin are capable of polarizing or maintaining DCs in the TME and draining lymph nodes in an immunosuppressive state, ultimately leading to decreased T cell activation and anti-tumor activity (89). Several additional studies have helped define an immune suppressive role for LILRB4 in cancer [reviewed in (90)].

LILRB4, LAIR-1 and OSCAR are all members of a larger Leukocyte Immunoglobulin-Like Receptor (LILR) family (91), several members of which have been described in cancer (92). It is interesting to speculate on how many additional members of this family of receptors, which are largely restricted to expression on immune subpopulations (93), interact with ECM proteins to stimulate or inhibit immunity in response to injured, remodeled, or dysregulated ECM ligands.

#### 3.1.6 CD44 and toll-like receptors

An example of a receptor capable of binding to multiple ECM constituents is CD44, a receptor expressed on immune and non-immune cells, whose extracellular domain contains binding sites for various ECM proteins including collagen, laminin, and fibronectin, although the primary functional ligand of CD44 is hyaluronic acid (HA) (9). Versican, another ECM constituent that also binds to CD44, can bind to PSGL-1 and Toll-Like Receptors (TLR) on immune cells, demonstrating the potential promiscuity of ECM protein interactions with multiple receptors (94).

TLRs are immune receptors that are critical for linking innate immune pathogen-sensing with adaptive immunity (95). However, reports have shown that TLR2 and TLR4 can interact with ECM-derived hyaluronan fragments as an endogenous danger signal to stimulate TLR signaling (96, 97), as well as the ECM proteoglycan components biglycan and lumican to stimulate cells in cancer (98, 99). TLR4 can also interact with Heparan Sulfate to trigger inflammatory cytokine production (100). Of course, TLRs and other immune receptors may interact with their primary ligands that are embedded in the ECM, or released from the ECM during collagen remodeling, adding to the complexity of how the ECM regulated immunity not only in cancer, but also other diseases (101).

As described above, current studies suggest that both inhibitory and stimulatory collagen receptors are prone to driving immune dysfunction in cancer. This may be attributable to the inherent dysregulation of collagen products in the TME that subsequently drives dysregulation of both stimulatory and inhibitory collagen receptor signaling in ways that synergize to disrupt TME anti-tumor immunity. While the list of ECM proteins that interact in some way with immune and non-immune receptors is extensive, the continued identification of immune receptors that specifically bind to collagen and non-collagen ECM core component ligands, and functionally regulate immune cell responses in cancer, will help build a more comprehensive understanding of the matrix-mediated immune suppression in cancer.

## 4 Novel disruptive strategies to overcome tumor collagen ECM-mediated immune suppression

### 4.1 Tumor ECM restricts the potential of immune checkpoint inhibitors

Immune checkpoint inhibitors (ICIs) have revolutionized how cancer is treated and how we think about cancer. ICIs can actively promote long-term, durable responses that may evolve with and continue to surveil cancer, resulting in curative outcomes, rather than a short-term extension of survival (102). ICIs for treating cancer have demonstrated widespread clinical success over the past decade by targeting PD-1 and CTLA-4 pathways (102). Nevertheless, a majority of patients are not responsive to approved immunotherapies (103). While many factors may be responsible for the lack of response, stromal dependent mechanisms are thought to play an important role (104). These include barrier function and immune exclusion that lead to so-called cold or excluded tumor types (71). While several more recent ICI therapeutics targeting LAG-3, TIM-3, TIGIT and others are currently in various stages of clinical trials or pre-clinical development, it is likely that these therapeutics will face the same limitations posed by stromal elements in a large percentage of patients, and cancer types, similar to the current limitations observed with approved ICIs (25, 26, 102).

The emerging link between cancer immunity and the ECM is based on active regulation of immune receptors by ECM core elements. As such, ECM mechanisms of action become blurred between barrier function in cold or excluded phenotypes, versus immunosuppressed phenotypes from, for example, LAIR-1 signaling, since mechanisms of action can and likely often overlap (71). Understanding the expanding universe of the ECM and how the various components restrict or promote immunity and immunotherapy in cancer needs to be dissected to optimize ICI and other immunotherapies. Studies evaluating collagen-derived peptides (CDPs) such as type III collagen pro-peptides (PRO-C3) in the serum of patients treated with PD-1 or CTLA-4 blockade have suggested an association with poor prognosis (105–108). PRO-C3 has been interpreted as being associated with dense fibrotic TME-ECM, but could also suggest tumor ECM remodeling (109). Emerging therapeutics that target multiple immune-ECM mechanisms of interaction, as well as combination strategies that synergize by targeting separate immune, TME and ECM components, will benefit patients who do not otherwise respond to existing therapies (110).

### 4.2 Therapeutics that target the intersection of the ECM and immune cell receptors

Several studies show that disrupting LAIR-1 interactions with collagens results in enhanced tumor immunity (55, 72, 77, 79, 111). Across these studies, it was demonstrated that therapeutic targeting of the LAIR-1 pathway in tumor models promoted the activation and function of T cells, NK cells, macrophages, and DCs. LAIR-2 is a soluble homolog of the transmembrane protein LAIR-1 that is present in human and non-human primate genomes, but not most other mammals, including mice (112). LAIR-2 binds to the same ligands as LAIR-1, but with higher affinity. It acts as a natural decoy protein in humans to block LAIR-1 interaction with collagen and downstream signaling, and possibly other collagen binding proteins (72, 112). This natural mechanism was taken advantage of to develop a novel therapeutic that would both target collagenous tumors and prevent LAIR-1 mediated signaling and adhesion. LAIR-2 fusion proteins generated in independent studies demonstrated anti-tumor activity of LAIR-2 Fc in multiple tumor models that was T cell dependent and modified the myeloid compartment (72, 77, 79). In other studies, *in vivo* overexpression of LAIR-2, or LAIR-1 blocking antibodies, were used to block LAIR-1 with demonstrable anti-tumor effects (55, 111). In studies with the collagen binding LAIR-2 IgG1 fusion protein, NC410, it was suggested that ECM remodeling may be occurring based on detection in changes in CDPs in the serum of NC410 treated mice (72). In support, specific collagen fragments of type IV collagen degraded by granzyme B (C4G) and type VI collagen degraded by MMP (C6M) has been shown *in vivo* to increase after induction of T cell activation by NC410 (72, 113). Additional studies have indicated that collagen degradative products are suppressive to T cells and therefore blockade of LAIR-1 and potentially other collagen receptor interactions with collagen degradative products may further normalize immune function in cancer (114).

LILRB4 blocking antibodies have demonstrated anti-tumor effects in solid tumor and hematological malignancies (115, 116). Additional modalities have also been developed and tested for targeting LILRB4 in cancer therapy, including antibody drug conjugates for direct cytotoxicity of LILRB4 expressing tumor associated myeloid cells, and LILRB4 CAR-T cells for targeting LILRB4 expressing leukemic cells (117, 118). Dasatinib (BMS-354825, Sprycel) is a small molecule Src inhibitor that non-specifically blocks DDR1/2 and other kinase receptors. It is used to treat chronic myelogenous leukemia and Philadelphia-positive acute lymphoblastic leukemia, but is also being tested in a wide range of solid tumors in a variety of combinations (reviewed in (119)). Recently, blocking DDR1 *in vivo* was also shown to reverse immune exclusion by disrupting collagen fiber alignment in breast cancer (120). A first-in-human study of this anti-DDR1 alone and in combination with anti-PD1 blockade has recently been initiated (NCT05753722). DDR2 inhibition in combination with PD-1 blockade has also demonstrated reduced tumor growth (121). Based on the accelerating interest in ECM binding immune receptors, the number of clinical trials targeting the LAIR, LILRB4, DDR1, DDR2 and other pathways will continue to expand from the current ongoing trials listed in **Table 1**.

**Table 1:** Clinical trials for LAIR-1, LILRB4 and DDR1 in solid and hematologic cancers.

Target	Drug	Format	Indication	Study	Identifier	Sponsor	Status
**LAIR-1**	NC410	LAIR-2 IgG1 fusion protein	Advanced or Metastatic Solid Tumors	A Phase 1/2, Open-Label, Dose-Escalation, Safety and Tolerability Study of NC410 in Subjects With Advanced or Metastatic Solid Tumors	NCT04408599	NextCure	Recruiting
**LAIR-1, PD-1**	NC410, Pembrolizumab	LAIR-2 IgG1 fusion protein	Advanced or Metastatic Solid Tumors	A Safety, Tolerability and Efficacy Study of NC410 Plus Pembrolizumab in Participants With Advanced Unresectable or Metastatic Solid Tumors	NCT05572684	NextCure	Recruiting
**LAIR-1**	NGM438, Pembrolizumab	N/A	16 tumor types	A Phase 1/1b Dose Escalation/Expansion Study of NGM438 as Monotherapy and in Combination With Pembrolizumab in Advanced or Metastatic Solid Tumors	NCT05311618	NGM Biopharmaceuticals, Inc	Active, not recruiting
**LAIR-1**	NC525	IgG1	Relapsed Refractory (R/R) AML, CMML, MDS	A Phase 1, Multicenter, Open-Label, Dose-Escalation and Expansion, Safety, Pharmacokinetic, Pharmacodynamic, and Clinical Activity Study of Intravenously Administered NC525	N/A	NextCure	Recruiting
**LILRB4**	IO-202, Azacitidine, Venetoclax	IgG4	AML With Monocytic Differentiation CMML	A Phase 1, Multicenter, Open-Label, Dose-Escalation and Expansion, Safety, Pharmacokinetic, Pharmacodynamic, and Clinical Activity Study of Intravenously Administered IO-202 and IO-202 + Azacitidine ± Venetoclax in Acute Myeloid Leukemia (AML) Patients With Monocytic Differentiation and in Chronic Myelomonocytic Leukemia (CMML) Patients	NCT04372433	Immune-Onc Therapeutics	Recruiting
**LILRB4**	IO-202, Pembrolizumab	IgG1	Solid Tumor, Adult	A Phase 1, Multicenter, Open-Label, Dose-Escalation, and Dose-Expansion Study of IO-202 in Combination With Pembrolizumab in Subjects With Advanced, Relapsed, or Refractory Solid Tumors	NCT05309187	Immune-Onc Therapeutics	Recruiting
**LILRB4**	LILRB4 STAR-T	chimeric antigen receptors (CAR) targeting cells expressing LILRB4	Relapsed/Refractory Acute Myeloid Leukemia	An Exploratory (Ph1) Clinical Study on the Safety and Efficacy of LILRB4 STAR-T Cells in the Treatment of Relapsed/Refractory Acute Myeloid Leukemia (R/R AML)	NCT05518357	Hebei Yanda Ludaopei Hospital	Completed
**LILRB4 and LILRB1**	NGM707, pembrolizumab	Bispecific mAb	15 tumor types	A Phase 1/2 Dose Escalation/Expansion Study of NGM707 as Monotherapy and in Combination with Pembrolizumab in Advanced or Metastatic Solid Tumor Malignancies	NCT04913337	NGM Biopharmaceuticals, Inc	Recruiting
**DDR1**	PRTH-101, pembrolizumab	N/A	Solid Tumors	A First-in-human Study of PRTH-101 Monotherapy +/- Pembrolizumab in Subjects With Advanced Malignancies	NCT05753722	Parthenon Therapeutics	Recruiting

N/A, Not available.

### 4.3 Therapeutics that target the ECM for immunotherapy combination strategies

Many attempts have been and continue to be made to target various ECM components in tumors to overcome drug resistance and for stroma normalization (122–124) Unfortunately, targeting these pathways alone has not been effective. Drugs that prevent the excess accumulation of ECM molecules are important for controlling tumor fibrosis, and altering the degradation of the ECM may be equally important for improving tumor immunity and immunotherapy, and eliminating tumor progression (45, 46, 125–127). It is proposed that targeting the ECM in combination with immunotherapies could synergize to activate immune cells and promote immune infiltration into the TME, while simultaneously disrupting other tumor promoting aspects of the ECM. Several studies have now indicated that therapeutic targeting of the collagen:LAIR-1 pathway in combination with PD-1 targeting therapies yields improved and synergistic activity in pre-clinical studies (55, 77). Additionally, a recent study that combines NC410 with Bintrafusp-alfa, a PD-L1 mAb fused with TGF-βRII demonstrated an even better outcome than NC410 with PD-1 blockade, suggesting the combination of checkpoint blockade and TME-ECM remodeling synergize to remodel immune responses in favor of anti-tumor immunity (79). This supports previous studies targeting TGF-β that have demonstrated improvement in anti-tumor immunity in combination with ICIs (128, 129).

LOX inhibitors have been shown to improve the response to PD-1 therapy (130). Along the same lines, modulating collagen expression and deposition in the tumor by targeting intracellular focal adhesion kinase (FAK) renders pancreatic cancers responsive to checkpoint inhibitor immunotherapy *in vivo* (131, 132). These studies validate the emerging therapeutic strategy of combining ECM targeting with immune checkpoint inhibitors for optimal activity and efficacy.

Importantly, collagen remodeling derived products (CDPs) are emerging as key players for defining the ECM and immune landscape of tumors and response to immunotherapy (133, 134). Such ECM protein biomarkers, ideally serum CDPs, may be identified to select indications and patients that are most likely to benefit from ECM-immune combination strategies (106, 108).

### 4.4 Directing and localizing therapeutics by targeting tumor specific ECM

Targeting therapeutics to and within tumors by targeting aberrant expression of ECM proteins in tumors is a growing strategy in cancer therapy. Conjugating PD-1 or CTLA-4 antibodies with ECM targeting agents, or fusing cytokines to ECM targeting agents for tumor localization have demonstrated positive pre-clinical results (135, 136). Anchoring of intratumorally administered cytokines to collagen safely potentiates systemic cancer immunotherapy (136). Cytokines have been fused with antibodies, or nanobodies, targeting specific domains of fibronectin (137). Fibronectin, Tenascin-C and other ECM proteins that are abundant in glioblastoma can be targeted for delivery of various payloads including RNA interference (138). These strategies and methodologies will undoubtedly be improved upon with advanced understanding of immune-ECM biology and may likely enter clinical testing soon.

**Figure 4:**
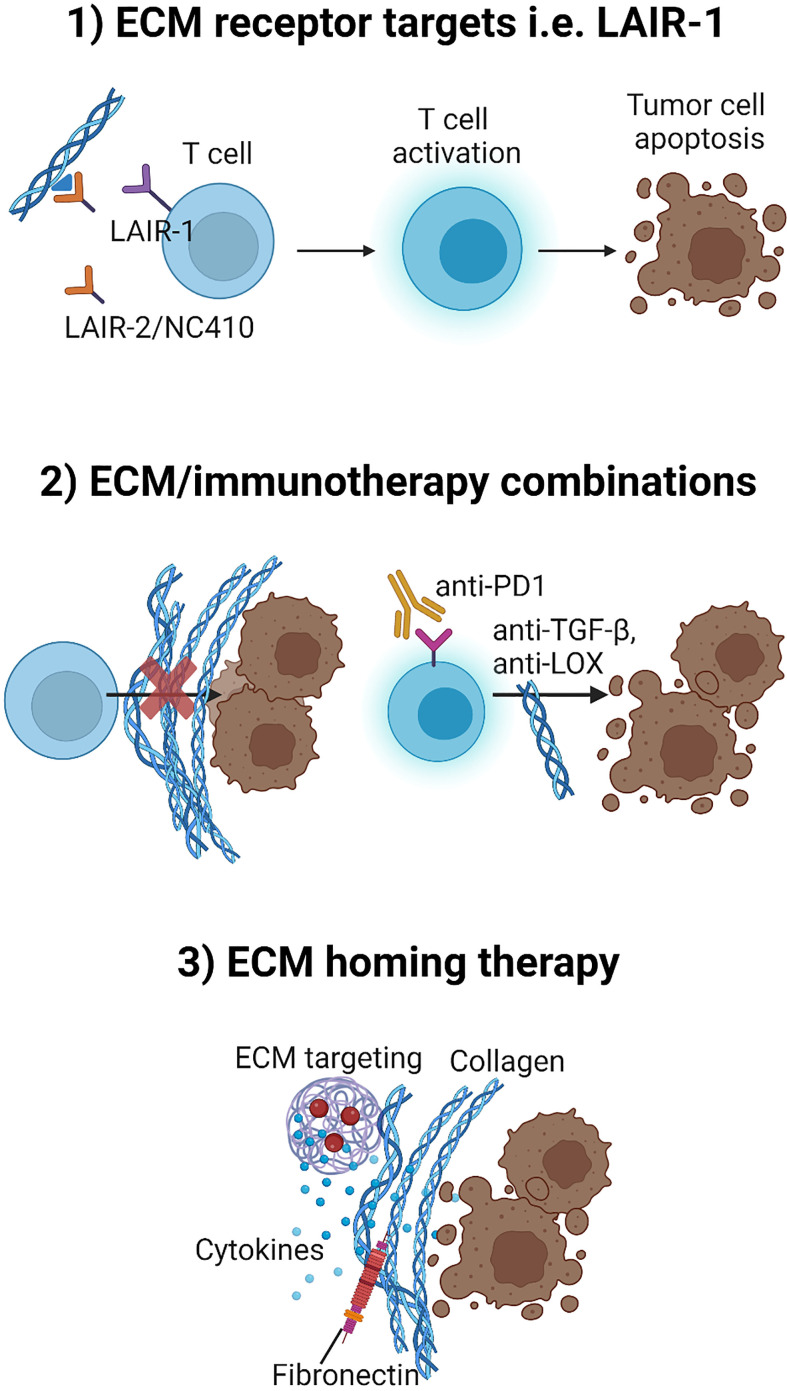
Three potential strategies to overcome tumor ECM/collagen-mediated immune suppression. 1) Developing therapeutics that targets ECM-immune cell receptors such as interfering with the immunosuppressive role of LAIR-1 by treatment with LAIR-2/NC410. 2) Developing combination strategies with therapeutics such as TGF-β inhibitors or LOX inhibitors that may target the tumor ECM for normalization and thereby improve efficacy of immunotherapy (anti-PD-1) combination strategies. 3) Directing and localizing therapeutics such as cytokines to the TME by fusing cytokines to ECM targeting agents for tumor localization. Figure created with BioRender.com.

The overall means of strategies described above to overcome tumor ECM/collagen-mediated immune suppression is shown in **Figure 4**.

## 5 Conclusion

Our expanded understanding in the fields of matrix biology, cancer biology, and immunobiology have contributed independently to improving upon the efficacy of cancer therapeutics. However, limited interaction and overlap has occurred to bring these fields together for developing novel cancer treatments. Expanding knowledge of the effects of collagen and other ECM components that actively regulate populations of immune cells in cancer will be important in helping to advance this emerging and exciting field of research. Importantly, it will also aid our ability to develop new classes of therapeutics to treat cancer and patients with unmet needs.

## Author contributions

All authors contributed equally to writing this manuscript.
